# Factors associated with low levels of aerobic fitness among adolescents

**DOI:** 10.1016/j.rppede.2015.06.025

**Published:** 2016

**Authors:** Eliane Cristina de Andrade Gonçalves, Diego Augusto Santos Silva

**Affiliations:** Programa de Pós-Graduação em Educação Física, Universidade Federal de Santa Catarina (UFSC), Florianópolis, SC, Brazil

**Keywords:** Association, Oxygen consumption, Lifestyle, Exercise, Adolescent health

## Abstract

**Objective:**

To evaluate the prevalence of low aerobic fitness levels and to analyze the association with sociodemographic factors, lifestyle and excess body fatness among adolescents of southern Brazil.

**Methods:**

The study included 879 adolescents aged 14-19 years the city of São José/SC, Brazil. The aerobic fitness was assessed by Canadian modified test of aerobic fitness. Sociodemographic variables (skin color, age, sex, study turn, economic level), sexual maturation and lifestyle (eating habits, screen time, physical activity, consumption of alcohol and tobacco) were assessed by a self-administered questionnaire. Excess body fatness was evaluated by sum of skinfolds triceps and subscapular. We used logistic regression to estimate odds ratios and 95% confidence intervals.

**Results:**

Prevalence of low aerobic fitness level was 87.5%. The girls who spent two hours or more in front screen, consumed less than one glass of milk by day, did not smoke and had an excess of body fatness had a higher chance of having lower levels of aerobic fitness. White boys with low physical activity had had a higher chance of having lower levels of aerobic fitness.

**Conclusions:**

Eight out of ten adolescents were with low fitness levels aerobic. Modifiable lifestyle factors were associated with low levels of aerobic fitness. Interventions that emphasize behavior change are needed.

## Resumo

**Objetivo:**

Avaliar a prevalência de baixos níveis de aptidão aeróbia e analisar sua associação com fatores sociodemográficos, estilo de vida e excesso de adiposidade corporal em adolescentes de uma cidade do sul do Brasil.

**Métodos:**

Estudo com 879 adolescentes de 14 a 19 anos de São José, SC, Brasil. A aptidão aeróbia foi avaliada pelo teste canadense modificado de aptidão aeróbia. Variáveis sociodemográficas (cor da pele, idade, sexo, turno de estudo, nível econômico), maturação sexual e estilo de vida (hábitos alimentares, tempo de tela, nível de atividade física, consumo de álcool e de tabaco) foram avaliados por questionário autoadministrado. O excesso de adiposidade corporal foi avaliado pelo somatório das dobras cutâneas do tríceps e subescapular. Empregou-se a regressão logística para a estimativa de *odds ratio* e intervalos de confiança de 95%.

**Resultados:**

A prevalência de baixo nível de aptidão aeróbia foi de 87,5%. As garotas que gastavam duas horas ou mais em frente à tela, que consumiam menos de um copo de leite ao dia, as não fumantes e com excesso de adiposidade corporal apresentaram mais chances de ter baixos níveis de aptidão aeróbia. Os garotos de cor de pele branca e que eram pouco ativos fisicamente apresentaram mais chances de ter baixo nível de aptidão aeróbia.

**Conclusões:**

Oito em cada dez adolescentes estavam com baixos níveis de aptidão aeróbia. Fatores modificáveis do estilo de vida foram associados com baixos níveis de aptidão aeróbia. Intervenções que enfatizem a mudança de comportamento são necessárias.

## Introduction

Secular trend studies have shown that the aerobic performance of young individuals is decreasing.^1^ The decrease has reached 0.36% a year,^1^ with a prevalence of low aerobic performance in approximately 80% among Brazilian adolescents.^2^

The high prevalence of inadequate levels of aerobic fitness in adolescents increases morbidity and mortality in adulthood, due to overweight,^3^ metabolic risk factors^3^ and cognitive diseases,^4^ in addition to causing difficulties in performing everyday activities.^5^ Conversely, the maintenance of adequate cardiopulmonary indexes, in itself, is capable of reducing health hazards and helping the recovery after intense physical exertion.^3^

The decline in aerobic fitness is associated with some individual characteristics, such as sociodemographic and life style factors.^6^ A systematic review identified the following factors associated with low levels of aerobic fitness: female gender, low economic level, lower consumption of dairy products and cereals, higher consumption of sweetened beverages, insufficient physical activity, excessive screen time and excess of body fat.^6^ However, it is controversial the association between low levels of aerobic fitness and other sociodemographic factors, such as skin color and age. Moreover, the association between aerobic fitness levels and excessive alcohol and tobacco consumption is scarcely studied.^6^

To assess the prevalence of low levels of aerobic fitness and possible related factors, such as sociodemographic and lifestyle indicators is justified because: (a) it contributes to the epidemiological knowledge of the subject; (b) it allows identifying whether the proportion of adolescents with insufficient levels of aerobic fitness is the same in different places; (c) it will allow effective interventions to be carried out at community and school level on the outcome. Therefore, the aim of this study was to evaluate the prevalence of low levels of aerobic fitness and to analyze the association with sociodemographic factors, lifestyle and excess body fat in high school adolescents living in a city in southern Brazil.

## Method

This cross-sectional analytical school-based study is part of the "Brazilian Guide of Physical Fitness Evaluation Related to Health and Life Habits - Stage I" macroproject (*Guia Brasileiro de Avaliação da Aptidão Física Relacionada à Saúde e Hábitos de Vida - Etapa I*). It was approved by the Institutional Review Board of Universidade Federal de Santa Catarina under protocol CAAE: 33210414.3.0000.0121, and was carried out between August and November 2014.

The population (*n*=5182) consisted of schoolchildren aged 14-19 years enrolled in public schools of São José, Santa Catarina (SC), Brazil, which has 209,804 inhabitants and a municipal Human Development Index of 0.809.^7^ São José shares borders with the state capital city of Florianópolis, and together they form the most densely populated metropolitan area of the state of SC. In addition to the fact that the school environment is a favorable place to encourage the implementation of a healthy and active lifestyle, as it is the place where young individuals spend much of their time, the choice of students from public institutions is justified because the schools and neighborhoods where they are located show social, cultural and economic discrepancies, which allows identifying adolescents with different cultures, ethnicities and lifestyles.

The sampling process was determined in two stages: 1 - stratified by state public high schools (*n*=11); 2 - class clusters considering the school shift and school year (*n*=170 classes). In Stage 2, all high school students who were present in classroom on the days of data collection were invited to participate in the study.

For sample size calculation, an unknown outcome prevalence rate was considered (50%), with a tolerable error of five percentage points, 95% confidence interval, design effect of 1.5; 20% were added for losses and refusals and an extra 20% for the association study. Thus, a sample of 751 adolescents was estimated. However, due to the cluster sampling, all students from all classes were invited to participate in the study, which resulted in 1148 students whose data were collected; 16 were excluded from the analysis because they were older than 19 years and thus, the final sample consisted of 1132 individuals.

The student eligible for the study was defined as: students enrolled in state schools, being present in the classroom on the data collection day and being 14-19 years. We considered a refusal when the adolescent declined to participate and, as sample loss, the students who returned an incomplete questionnaire or did not undergo one or more physical tests.

Aerobic fitness was measured using the modified Canadian Aerobic Fitness Test (mCAFT),^8^ validated in comparison with indirect calorimetry in Canadian men and women aged 15-69 years.^9^ The adolescents had to complete one or more stages lasting three minutes each (go up and down two steps measuring 20.3cm each) at pre-determined rhythms according to the gender and age. The test was terminated only when the participant reached 85% of maximum heart rate (recommended by the formula 220-age),^8^ which was measured using a Polar^®^ H7 Bluetooth frequency meter. For adolescents who completed at least one stage, but stopped in the middle of the other, the previous stage was recorded as the final stage.

Oxygen consumption and the reference values of aerobic fitness were determined using the Canadian tests.^8^ The aerobic fitness score equation is:


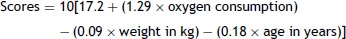


Based on this score, each participant was classified into one of the following five categories: (a) "Needs Improvement"; (b) "Fair"; (c) "Good"; (d) "Very good"; (e) "Excellent". In this study, aerobic fitness was considered "normal/high" for adolescents in categories (c), (d), (e) and "low" in categories (a) and (b), as the aim of this study was to identify the subgroups that were more likely to show low levels of aerobic fitness, which result in several problems for the adolescents' health and allow the development of different diseases in adulthood.^1^^,^ 3 ^,^ 4

The sociodemographic and lifestyle variables were collected through a self-administered questionnaire. The questions about eating habits, physical activity, excessive consumption of alcohol and tobacco were taken from the Youth Risk Behavior Survey questionnaire, translated and validated for Brazil.^10^

Skin color was self-reported according to the Brazilian Institute of Geography and Statistics^11^ and dichotomized into "White" and "Mixed-race/Black/Yellow/Native Brazilian". Age was categorized as "14-16 years" and "17-19 years". The economic level was identified by Abep^12^ and dichotomized into "high" ("A1"; "A2"; "B1"; "B2") and "Low" ("C1"; "C2"; "D"; "E"). The school shift was categorized as "day shift" (morning, afternoon or full-time) and "night shift" (night time).

Analysis of the screen time was carried out by applying six different questions, which checked the amount of hours spent watching television, using the computer and video game, weekly and on weekends. The screen time was calculated by the sum of hours of screen time on weekdays (calculated by multiplying the hours and minutes by five) and weekend (calculated by multiplying the hours and minutes by two), which resulted in the total screen time. The daily mean hours was verified by the sum of hours on the seven days of the week, divided by the days of the week (seven days) for the three types of electronic devices (television, computer and video games). The categories were "appropriate" (<2h per day) and "inappropriate" (≥2h daily).^13^

Eating habits were analyzed by two different questions about how many times the adolescent consumed soft drinks and milk during the last seven days prior to the survey. The categories were: "Adequate" (adolescent who did not consume soft drinks); "Inadequate" (adolescent that consumed soft drinks)^14^ and "Adequate" (consumed ≥1 glass of milk/day); "Inadequate" (Consumed <1 glass/day).^15^ We decided to analyze these two indicators because a study identified a reduction in milk consumption and an increase in soft drink consumption in Brazil.^15^ This situation has a negative impact on the health of young individuals, and causes, for instance, bone density impairment due to the low consumption of milk, which can decrease performance in physical activity and the increase in overweight/obesity due to consumption of soft drinks.^15^

The question regarding physical activity was: "During the past seven days, how many days were you physically active for at least 60min a day?" The adolescents who practiced physical activity five or more days/week were classified as "physically active (≥300min a week)" and less than five days/week as "insufficiently physically active (<300min per week)".^16^^,^ 17

The questions about smoking and excessive alcohol consumption were based on the number of days (30 days prior to the study) in which the adolescent smoked and consumed five or more alcoholic drinks on a single occasion. The categories were: "No" (did not smoke); "Yes" (smoked a day or more),^18^ "No" (did not consume five or more alcoholic drinks on a single occasion); "Yes" (consumed five or more alcoholic drinks on a single occasion).^19^

Excess body fat was measured through two skinfolds (triceps and subscapular) using a Cescorf^®^ caliper, according to the standardization of the International Society for the Advancement of Kinanthropometry (ISAK). Anthropometric measurements were obtained by a single evaluator with a level 1 ISAK certification. The results of the skinfolds were added and analyzed according to Lohman,^20^ taking gender into account. Adolescents with a sum ≥30mm and ≥35mm, for boys and girls respectively, were classified as having excess body fat.

Sexual maturation was assessed according to the criteria proposed by Tanner,^21^ validated and reproducible in the Brazilian population.^22^ The indication of pubertal stages was performed by self-assessment (figures) of breast development (females) and genitals (males). This variable was dichotomized into "Pre-pubertal/pubertal" and "Post-pubertal", as the sample had a low frequency of pre-pubertal adolescents.

The descriptive analysis of the variables used means, standard deviations and frequency distribution. Data normality was verified through sampling distribution histograms; however, no variable showed normal distribution. The Chi-square test of heterogeneity was applied to identify differences in the prevalence of low levels of aerobic fitness according to the independent variables.

Binary logistic regression was employed and the odds ratio (OR) and 95% confidence interval were estimated. All variables were controlled for sexual maturation and introduced in the adjusted model irrespective of the *p*-value in the crude analysis. The analysis was performed hierarchically,^23^ divided into three blocks: 1 - demographic factors (Distal); 2 - economic level and school shift (Intermediate 1); 3 - lifestyle (Intermediate 2) and 4 - excess body fat (Proximal). The variables with *p*-value <0.20^24^ remained in the adjusted model when the backward adjusted analysis was performed. The significance level was set at 5%. The analysis were performed using the Statistical Package for Social Sciences (SPSS) version 22.0, considering the effect of design and sample weight and are shown for the entire sample and stratified by gender.

## Results

Of the 1132 analyzed students, 253 were excluded from the analysis for not undergoing the aerobic fitness test, which resulted in 879 students. Tables 1 and 2 show the characteristics of the sample.

**Table 1:** Total and stratified values by gender of the mean and standard deviation of age, anthropometric variables, screen time and aerobic score.

	Total sample	Male	Female	*p*-value
Age	16.2±1.1	16.2±1.1	16.1±1.1	0.15
Body mass (kg)	61.7±12.2	65.4±12.0	58.3±11.3	0.25
Height (cm)	166.6±8.8	172.5±7.3	161.1±6.0	<0.01^a^
Screen time (h)	6.5±4.9	7.0±4.9	5.9±4.8	<0.01^a^
Triceps	14.9±7.3	10.7±5.1	18.7±6.9	<0.01^a^
Subscapular	13.3±6.7	10.7±4.8	15.6±7.3	<0.01^a^
Sum of folds (mm)	28.2±13.4	21.5±9.5	34.3±13.6	<0.01^a^
Aerobic score	388.1±58.3	426.8±53.4	353.3±36.6	<0.01^a^

M, mean; SD, standard deviation. Sum of triceps and subscapular skin folds.

a *p*≤0.05 (Mann-Whitney test).

**Table 2:** Distribution of the total and stratified sample by gender in relation to sociodemographic factors, lifestyle, excess of body fat, sexual maturation and level of aerobic fitness.

Variables	Total sample	Male	Female
	*n* (%)	*n* (%)	*n* (%)
White skin color	541 (62.4)	253 (62.2)	288 (62.6)
14-16 years old	506 (57.6)	232 (55.9)	274 (59.1)
High economic level	503 (67.8)	255 (73.9)	248 (62.5)
Daytime school shift	623 (71.5)	287 (70.0)	336 (72.9)
Inadequate screen time	710 (85.4)	352 (90.3)	358 (81.2)
Soft drink consumption	731 (84.2)	355 (87.0)	376 (81.7)
Inadequate milk consumption	616 (70.6)	283 (68.7)	333 (72.2)
Insufficiently physically active	653 (76.4)	285 (70.7)	368 (81.4)
Cigarette smoking	65 (7.5)	28 (6.8)	37 (8.1)
Alcohol consumption	285 (32.6)	129 (31.3)	156 (33.8)
Excess of body fat	231 (26.3)	50 (12.1)	181 (39.1)
Prepubertal/pubertal	627 (71.8)	308 (74.9)	319 (69.0)
Low level of aerobic fitness	769 (87.5)	354 (85.3)	415 (89.4)

The prevalence of low aerobic fitness was 87.5% (85.3% of boys and 89.4% girls). Adolescents who were insufficiently active had two or more hours of screen time, had excess body fat and higher prevalence of low levels of aerobic fitness (*p*<0.05) (Table 3). Boys of white ethnicity, insufficiently active and with excess body fat had a higher prevalence of low levels of aerobic fitness. Girls that had two or more hours of screen time, did not smoke and had excess body fat had a higher prevalence of low levels of aerobic fitness (*p*<0.05) (Table 3).

**Table 3:** Sample distribution in relation to sociodemographic significant factors, lifestyle, sexual maturation and excess of body fat associated with the level of aerobic fitness.

	Aerobic fitness - total				Aerobic fitness - male gender				Aerobic fitness - female gender		
	Normal/high	Low			Normal/high	Low			Normal/high	Low	
	*n* (%)	*n* (%)	*p*-value		*n* (%)	*n* (%)	*p*-value		*n* (%)	*n* (%)	*p*-value
*Skin color*			0.58				0.04^a^				0.64
White	59 (10.9)	482 (89.1)			30 (11.9)	223 (88.1)			29 (10.1)	259 (89.9)	
Mixed race/Black/Yellow/Native Brazilian	50 (15.3)	276 (84.7)			30 (19.5)	124 (80.5)			20 (11.6)	152 (88.4)	
											
*Level of physical activity*			0.01^a^				0.04^a^				0.32
Physically active	36 (17.8)	166 (82.2)			24 (20.3)	94 (79.7)			12 (14.3)	37 (10.1)	
Insufficiently physically active	72 (11.0)	581 (89.0)			35 (12.3)	250 (87.7)			37 (10.1)	331 (89.9)	
											
*Screen time*			0.02^a^				0.34				0.01^a^
Adequate	23 (19.0)	98 (81.0)			08 (21.1)	30 (78.9)			15 (18.1)	68 (81.9)	
Inadequate	82 (11.5)	628 (88.5)			52 (14.8)	300 (85.2)			30 (8.4)	328 (91.6)	
											
*Excess of adiposity*			**<**0.01^a^				<0.01^a^				<0.01^a^
No	104 (16.1)	542 (83.9)			61 (16.8)	303 (83.2)			43 (15.2)	239 (84.8)	
Yes	06 (2.6)	225 (97.4)			00 (0.0)	50 (100.0)			06 (3.3)	175 (96.7)	

Excess of adiposity=excess of body fat. Physically active ≥300min/week.

a *p*<0.05 (Chi-square test).

The crude analysis showed that adolescents of white ethnicity that two or more hours of screen time, who consumed alcohol in excess and had excess body fat were more likely to have low levels of aerobic fitness (Table 4). In the adjusted analysis, the adolescents who spent two hours or more in front of the screen, had inadequate consumption of milk, were insufficiently active and had excess body fat were more likely to have low levels of aerobic fitness (Table 4).

**Table 4:** Crude and adjusted logistic regression analysis between low aerobic fitness and sociodemographic factors, lifestyle and excess of body fat in adolescents.

Variables	Total sample			Male			Female	
	Crude analysis	Adjusted analysis		Crude analysis	Adjusted analysis^a^		Crude analysis	Adjusted analysis^a^
	OR (95%CI)			OR (95%CI)			OR (95%CI)	
Non-white skin color	0.7 (0.5-0.9)^a^	0.7 (0.5-1.0)		0.6 (0.3-0.9)^a^	0.6 (0.3-0.9)^a^		0.9 (0.5-1.6)	0.8 (0.5-1.5)
Inadequate screen time	1.8 (1.1-3.0)^a^	1.7 (1.1-3.0)^a^		1.5 (0.7-2.0)	1.3 (0.5-3.3)		2.2 (1.1-4.3)^a^	2.3 (1.1-4.5)^a^
Inadequate milk consumption	1.7 (1.1-2.6)^a^	1.7 (1.1-2.6)^a^		1.2 (0.7-2.1)	1.1 (0.6-2.1)		1.6 (0.8-2.9)	2.0 (1.1-3.9)^a^
Insufficiently physically active	1.6 (1.1-2.5)^a^	1.6 (1.1-2.4)^a^		1.8 (1.1-3.2)^a^	2.0 (1.1-3.7)^a^		1.5 (0.7-3.0)	1.5 (0.7-3.2)
Alcohol consumption	0.8 (0.5-1.2)	0.8 (0.5-1.3)		0.6 (0.3-1.1)	0.7 (0.4-1.2)		0.7 (0.4-1.3)	1.1 (0.5-2.1)
Cigarette smoking	0.6 (0.3-1.1)	0.5 (0.2-1.1)		1.1 (0.3-3.1)	1.2 (0.4-4.0)		0.4 (0.2-0.9)^a^	0.4 (0.1-0.8)^a^
Excess of body fat	7.9 (3.1-20.2)^a^	7.9 (3.19-20.1)^a^		32.5 (27.6-40.0)^a^	35.5 (26.0-45.0)^a^		5.2 (2.1-12.6)^a^	6.1 (2.3-16.2)^a^
Post-pubertal	1.1 (0.6-1.7)	1.1 (0.6-1.8)		1.4 (0.7-2.8)	1.6 (0.8-3.2)		1.1 (0.5-2.1)	0.9 (0.4-1.9)

OR, odds ratio; CI, confidence interval. Analysis adjusted for all the variables, with control by the sexual maturation, regardless of *p*-value in the crude analysis.

a *p*<0.05.

In both crude and adjusted analyses, boys of white ethnicity that were insufficiently active were more likely to have low levels of aerobic fitness. In the crude analysis, the girls who did not smoke and had excess body fat were more likely to have low levels of aerobic fitness (Table 4). In the adjusted analysis, girls that had two or more hours of screen time and had an inadequate consumption of milk were more likely to have low levels of aerobic fitness (Table 4).

## Discussion

The prevalence of low levels of aerobic fitness in this study was similar to that found in a study carried out in five Brazilian regions with 7057 children and adolescents.^2^ The girls in this study had a higher prevalence of low levels of aerobic fitness than boys. This fact is justified because girls practice less physical activity during adolescence and have lower left ventricular mass when compared with boys, which determines lower systolic volume at rest and results in lower aerobic performance.^25^

For the total sample and for the female gender, the adolescents that had two or more hours of screen time showed low levels of aerobic fitness. Similar results were found in a study carried out in the United States.^3^ That occurs because during the time spent with electronic devices, adolescents fail to perform more intense activities, which results in low levels of physical activity and physical fitness in general.^5^

Low levels of aerobic fitness were associated with inadequate consumption of milk in the total sample and for the female gender, which is similar to the study carried out in seven European countries with adolescents aged 12-17 years.^26^ This finding is of concern, as milk is a source of protein, amino acids, vitamins and carbohydrates (lactose), being used as energy by muscles.^15^^,^ 26 Additionally, milk nutrients are responsible for the reestablishment of fluid balance after exercise-induced dehydration, and aid in muscle mass gain.^15^^,^ 26 Thus, the inadequate consumption of milk influences physical performance in childhood and adolescence.^26^

In the total sample and for the female gender, the adolescents with excess body fat were nearly eight times more likely to have low levels of aerobic fitness. Other studies have also found an association between these variables.^3^^,^ 25 A possible justification is that individuals with high levels of body fat are more likely to have difficulties in locomotion, which influences in the economy of movement, greater energy consumption and early fatigue in aerobic activities, which decreases performance in physical tests.^6^

The girls who did not smoke had low levels of aerobic fitness. This finding may be an association known as a false one, due to systematic or random errors, inherent to epidemiological studies based on empirical observations from samples.^27^ Systematic errors can make an association that actually does not exist look real.^27^ Additionally, the systematic review identified that there are no investigations on low aerobic fitness and smoking in adolescents, which emphasizes the need for studies to investigate the association between these two variables.^6^ What the literature affirms is that, although the individual might have a good level of physical activity or physical fitness, smoking alone impairs the performance and general health.^28^

For the total sample and for the male gender, the adolescents who were insufficiently active had low aerobic fitness levels, similar to the results found in a study carried out in Spain with adolescents aged 12-18 years.^25^ The association between these variables occurs because the insufficient physical activity practice or low-intensity activities are insufficient to meet the required threshold for cardiovascular adaptations that increase aerobic fitness levels.^3^^,^ 25

The white ethnicity in boys showed low levels of aerobic fitness. This fact is justified because white ethnicity adolescents usually constitute the most favored economic classes.^29^ Thus, they have greater access to convenience and fast food stores, TV, computers and video games, which can increase the time spent in sedentary activities.^29^

The study limitations were: 1 - the fact that the adolescents know they are participating in a study on lifestyle can, in itself, have influenced the low prevalence results, especially regarding the excessive consumption of alcoholic beverages and cigarettes; 2 - as it was not possible to control the adolescents' movements before the aerobic fitness test, some individuals may have initiated the test with a heart rate above the resting heart rate.

This investigation contributes to the area of study, as it shows different sociodemographic and lifestyle variables, chosen in order to disclose a greater overview of possible factors correlated to the low aerobic fitness levels in adolescents and contributes to the analysis and discussion of modifiable sociocultural and behavioral aspects that influence the low aerobic performance. Additionally, it discloses data on the aerobic fitness level among adolescents from a city in southern Brazil, serving as a comparative parameter for studies with young individuals. Moreover, the association between low levels of aerobic fitness and sociodemographic factors, lifestyle and excess of body fat highlights the need for planning programs to improve the aerobic performance of students, aiming to reduce the health risks generated by this condition.

The conclusion is that eight of ten adolescents had inadequate levels of aerobic fitness for good health. The white ethnicity and insufficiently active boys and nonsmoker girls that had excess of body fat, had two or more hours of screen time and had inadequate milk consumption were more likely to have low levels of aerobic fitness.
